# Epidemiology of Multidrug-Resistant Pseudomonas aeruginosa in the Middle East and North Africa Region

**DOI:** 10.1128/mSphere.00202-21

**Published:** 2021-05-19

**Authors:** Mahmood Al-Orphaly, Hamad Abdel Hadi, Faiha Kamaleldin Eltayeb, Hissa Al-Hail, Bincy Gladson Samuel, Ali A. Sultan, Sini Skariah

**Affiliations:** a Department of Medical Education, Weill Cornell Medicine - Qatar, Education City, Qatar Foundation, Doha, Qatar; b Department of Infectious Diseases, Communicable Diseases Centre, Hamad Medical Corporation, Doha, Qatar; c Department of Laboratory Medicine and Pathology, Hamad Medical Corporation, Doha, Qatar; d Department of Microbiology and Immunology, Weill Cornell Medicine - Qatar, Education City, Qatar Foundation, Doha, Qatar; Escola Paulista de Medicina/Universidade Federal de São Paulo

**Keywords:** *Pseudomonas aeruginosa*, antibiotic resistance, multidrug resistance, Middle East and North Africa region, intensive care units, urinary tract infections, MDR, MENA

## Abstract

Over the last decades, there has been a dramatic global increase in multidrug-resistant (MDR) pathogens particularly among Gram-negative bacteria (GNB). Pseudomonas aeruginosa is responsible for various health care-associated infections, while MDR P. aeruginosa causes significant morbidity and mortality. Middle East and North Africa (MENA) represent an unexplored geographical region for the study of drug resistance since many of these countries are at crossroads of high volume of travel, diverse expatriate populations, as well as high antibiotic consumption despite attempts to implement antimicrobial stewardship programs. This minireview analyzes epidemiology, microbiological, and genomic characteristics of MDR P. aeruginosa in the MENA region. Published data on MDR P. aeruginosa prevalence, antimicrobial resistance patterns, and genetic profiles from studies published during the past 10 years from 19 MENA countries have been included in this minireview. There is wide variation in the epidemiology of MDR P. aeruginosa in the MENA region in terms of prevalence, antimicrobial characteristics, as well as genetic profiles. Overall, there is high prevalence of MDR P. aeruginosa seen in the majority of the countries in the MENA region with similarities between neighboring countries, which might reflect comparable population and antibiotic-prescribing cultures. Isolates from critical care units are significantly resistant particularly from certain countries such as Saudi Arabia, Egypt, Libya, Syria, and Lebanon with high-level resistance to cephalosporins, carbapenems, and aminoglycosides. Colistin susceptibility patterns remains high apart from countries with high-level antibiotic resistance such as Saudi Arabia, Syria, and Egypt.

## INTRODUCTION

Over the past decades, there has been a remarkable global increase in antimicrobial resistance (AMR). A report published by the Centers for Disease Control and Prevention in 2019 stated that these pathogens are responsible for the annual infection of more than 2.8 million people and cause an estimated 35,000 deaths per year in the United States alone (1). In the European Union, infections with AMR pathogens cause approximately 33,000 deaths per year with an estimated annual economic loss of 1.5 billion dollars (2). Furthermore, it has been estimated that by 2050 if no action has been taken, mortality and morbidity from AMR will surpass any acute or chronic illnesses, including heart diseases and cancer with an estimated annual mortality of 10 million cases (2, 3). The escalated challenge caused by AMR pathogens extends beyond developing countries to include the Middle East and North Africa (MENA) region which has not been fully explored.

Of particular concern of the global propagation of AMR is the continuous emergence of multidrug-resistant (MDR) pathogens particularly in Gram-negative bacteria (GNB). Among GNB, members of the *Enterobacteriaceae* family, Pseudomonas aeruginosa, and Acinetobacter baumannii are considered major health care threats due to their vast rates of emergence, acquisition, and spread of different resistance mechanisms (4). This consequently led to challenges in initiating appropriate targeted therapy particularly in severe infections leading to increased morbidity and mortality as well as prolonged hospital stays and subsequently excessive health care costs (5). To counter these challenges, broad-spectrum antimicrobials such as advanced cephalosporins and carbapenems have been overprescribed repeatedly, leading to a vicious cycle with further accumulation of selective resistance profiles (6).

P. aeruginosa is an opportunistic GNB which was first identified in the early 1800s (7). It thrives best in moist settings particularly suitable aquatic environments, including health care settings (8). Despite widespread environmental spread, P. aeruginosa rarely colonizes healthy individuals (0% to 2% skin colonization rate) (8). However, it frequently colonizes hospitalized patients (>50% colonization rate) and is a major cause of health care-associated infections (HCAIs) leading to life-threatening acute or chronic infections, including recurrent exacerbations in patients with cystic fibrosis, hospital- and ventilator-associated pneumonia, bacteremia, urinary tract, as well as wounds and soft tissue infections (8, 9).

While studying the underlying resistance mechanism of AMR, P. aeruginosa is a cornerstone pathogen being highlighted among the important resistant ESKAPE bacteria (Enterococcus faecium, Staphylococcus aureus, Klebsiella pneumoniae, Acinetobacter baumannii, P. aeruginosa, and Enterobacter species) which are the foremost challenging pathogens for community and hospital drug resistance (10). It is also considered a critical priority by the WHO’s ranking list of pathogens in need to develop and discover novel therapeutic modalities (11).

The Middle East and North Africa region represents a comprehensively uncharted geographical region for drug resistance studies, since many of these countries have high volume of travel, diverse expatriate populations, and wide availability of over-the-counter antibiotics despite recent attempts to control antimicrobial consumption through antimicrobial stewardship programs (ASPs) at community and hospital levels (12–16). Although there are published reports on the epidemiology of P. aeruginosa from individual countries, there has been no comprehensive review covering the entire region to the best of our knowledge. The aim of this minireview is to describe the prevalence of MDR P. aeruginosa, microbiological characteristics, and genomic mechanisms of antibiotic resistance focusing mainly on carbapenem resistance in the Middle East and North Africa region.

## MECHANISMS OF ANTIMICROBIAL RESISTANCE IN GNB INCLUDING P. AERUGINOSA

There are multiple antimicrobial mechanisms that evolved in Gram-negative bacteria (GNB) to become distinctively resistant. Integral to its resistance mechanisms is the production of *β*-lactamases which disrupt the *β*-lactam rings of antibiotics that target bacterial cell walls (4). All *β*-lactam antibiotics contain the 3-carbon and 1-nitrogen ring (beta-lactam ring), which includes commonly prescribed antibiotic classes such as penicillins, monobactams, cephalosporins, as well as carbapenems (17). According to molecular size and amino acid similarity in the active sites, *β*-lactamases are subdivided into molecular classes A through D (Ambler classification), and accumulation of diverse *β*-lactamase resistance genes manifest as highly resistant strains (18). Extended-spectrum *β*-lactamases (ESBLs) (which are mainly class A *β*-lactamases) are the most commonly encountered, leading to resistance to all *β*-lactams, except carbapenems and certain *β*-lactam *β*-lactamase inhibitor combinations (BLBLIs) (19). Conversely, metallo-*β*-lactamase (MBL) (zinc-based class B) cause resistance to all *β*-lactams, including BLBLIs except monobactams such as aztreonam. Because metallo-*β*-lactamases are encoded by mobile gene cassettes, they are often associated with other resistance genes such as aminoglycosides and fluoroquinolones (4). Similarly, AmpC (class C) are broad-spectrum *β*-lactamases with cephalosporinase selection preference (9), while oxacillinases (class D) are clinically relevant *β*-lactamases capable of hydrolyzing the potent class of carbapenems, raising the height of resistance profiles (4). The most common *β*-lactamases in P. aeruginosa are class A (VEB, PME, BEL, GES, and PER), class B (VIM and IMP), and class D (OXA-2 and OXA-10) *β*-lactamases (20).

In addition to *β*-lactamases, GNB, particularly P. aeruginosa, possess other corresponding resistance mechanisms which are either intrinsically present at the chromosomal level or horizontally acquired through plasmids demonstrated by downregulation of porin channels, upregulation of efflux pumps, antibiotic modification, as well as target site alteration (21). In P. aeruginosa, the main chromosomally encoded intrinsic mechanisms are inducible AmpC cephalosporinase, MexAB-OprM efflux pumps, inducible MexXY efflux pump, as well as low outer membrane permeability and OXA-type oxacillinase (20). In addition to its naturally occurring intrinsic resistance mechanisms, P. aeruginosa is capable of accumulating resistance genes acquired via chromosomal mutations (21). Overproduction of chromosomal AmpC cephalosporinase is likely the most prevalent acquired *β*-lactam resistance mechanism, and it has been found in over 20% of clinical isolates (20, 22–24). Structural modification of AmpC is another potential cause of *β*-lactam resistance (25, 26). Inducible AmpC causes resistance to aminopenicillins (e.g., amoxicillin and ampicillin) and a number of cephalosporins (particularly cefoxitin) (20). Overexpression of efflux pumps can also be affected by chromosomal mutations. Overexpression of the MexAB-OprM efflux pump allows resistance to most of the *β*-lactams (except imipenem) and fluoroquinolones, and inducible MexXY efflux pump overexpression allows resistance to aminoglycosides, cefepime, and chloramphenicol (20). Although less common, MexCD-OprJ and MexEF-OprN overexpression can cause resistance to fluoroquinolones (20, 27, 28). In addition, inactivation or downregulation of the carbapenem-specific porin OprD can cause acquired resistance to imipenem and decreased susceptibility to meropenem (22, 29). Together, AmpC overproduction and OprD inactivation can potentially cause resistance to all antipseudomonal *β*-lactams (20, 30). Other mutation-driven resistance mechanisms that can be acquired are via mutations in DNA gyrases (GyrA/GyrB) and type IV topoisomerases (ParC/ParE), both of which cause resistance to fluoroquinolones (8, 31). Additionally, mutations in FusA1 can cause resistance to aminoglycosides (28, 32). Last, among mutation-driven mechanisms, alteration, or modifications of the outer membrane liposaccharide (LPS) operon might generate resistance to colistin (20, 33).

Of note, horizontally transferred resistance is yet another method of acquiring esistance in P. aeruginosa (20). This is mostly seen in acquiring ESBLs and carbapenemases, specifically of class A serine carbapenemases (KPC), class B (metallo-*β*-lactamases), and class D (OXA-40) (9). The genes encoding these *β*-lactamases are found on class 1 integrons, which are inserted into mobile elements and mediate transfer between bacteria (34, 35). Horizontally transferred plasmid-mediated colistin resistance genes that disrupt outer LPS have been infrequently reported in P. aeruginosa (36). In addition, the class 1 integrons also carries determinants of aminoglycoside resistance, mainly acetyltransferases and nucleotidyltransferases (9, 37). Transferrable fluoroquinolone resistance has also been reported in P. aeruginosa (38).

Despite extensive regional reporting of the epidemiology and microbiological and genomic characteristics of P. aeruginosa, including resistant strains, limited similar information is available regarding the Middle East and North Africa (MENA) region. This minireview aims to explore available literature for the scale of the problem and report the microbiological and genetic characteristics of dominant strains in the MENA region, including highly resistant strains such as carbapenem-resistant P. aeruginosa.

## METHODS AND DATA COLLECTION AND ANALYSIS

**FIG 1:**
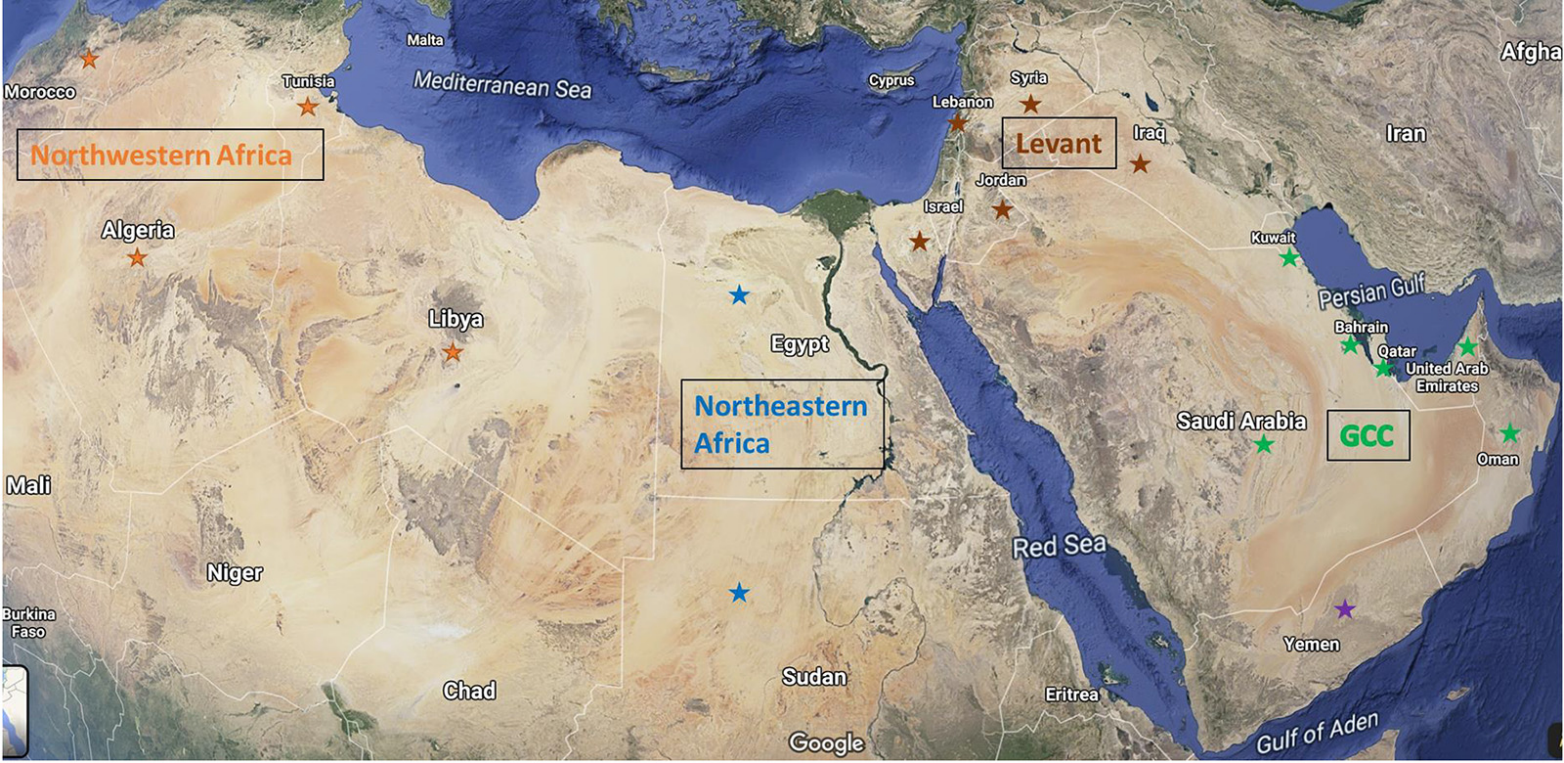
Google map image of the MENA region highlighting the countries included in this minireview. Imagery ©2021 TerraMetrics; map data ©2021 Mapa GISrael, Google.

Four clusters of the MENA region, encompassing 19 countries, were included in this study: the Levant region (Iraq, Syria, Lebanon, Palestine, Israel, and Jordan), Gulf Countries comprising the Gulf Cooperation Council (GCC) (Saudi Arabia, Kuwait, Qatar, United Arab Emirates [UAE], Bahrain, and Oman) in addition to Yemen, northeastern Africa (Egypt and Sudan), and northwestern Africa (Libya, Tunisia, Algeria, and Morocco) (Fig. 1). The PubMed database was searched for studies from the MENA region that report MDR rates in P. aeruginosa, antimicrobial susceptibility/resistance profiles, as well as reported resistance genes in carbapenem-resistant (CR) P. aeruginosa. Antibiotics and genes most reported and clinically relevant have been included in this minireview. Studied antibiotics are BLBLI (piperacillin-tazobactam [PTZ]), third-generation cephalosporin (ceftazidime [CTZ]), fourth-generation cephalosporin (cefepime [FEP]), carbapenems (imipenem [IMP] and meropenem [MER]), aminoglycosides (amikacin [AMK] and gentamicin [GEN]), fluoroquinolones (ciprofloxacin [CIP] and levofloxacin [LEV]), monobactam (aztreonam [AZT]), and colistin (COL).

The examined genes of interest are as follows: class A ESBL genes (*bla*_SHV_, *bla*_GES_, *bla*_TEM_, *bla*_KPC_, *bla*_CTX-M_, and *bla*_VEB_); class B metallo-*β*-lactamases (MBL) genes (Verona integron-encoded MBL [*bla*_VIM_], imipenemase MBL [*bla*_IMP_], New-Delhi MBL [*bla*_NDM_]); class C genes (*ampC*); class D oxacillinase genes (*bla*_OXA_), as well as the *oprD* gene and *mex* efflux pump genes (*mexAB-oprM*, *mexCD-oprJ*, *mexEF-oprN*, and *mexXY*). Overexpression of the efflux pumps MexAB, MexCD, MexEF, and MexXY was considered when transcription/protein of either components of the MexAB, MexCD, MexEF, or MexYZ are reported to be upregulated, respectively.

For the purpose of this minireview, for each country when data are available, P. aeruginosa isolates have been stratified based on their source: general clinical (GC) samples (mix of samples from various clinical units), intensive care unit (ICU) samples (reported studies primarily consisting of samples from intensive and critical care units), and urinary tract infection (UTI) samples. For each of the stratifications used in the minireview, the available most recent scientific papers from each country published within the past 10 years were included. Data from a total of 60 research papers are included in this minireview. The prevalence of MDR P. aeruginosa reflects reported rates of MDR P. aeruginosa out of the total P. aeruginosa infections. When reported, the standard definition of MDR P. aeruginosa has been generally adopted as resistance to at least one agent from three different classes as endorsed by agreed international consensus (39). Furthermore, this minireview describes regional variations in antibiotic susceptibility and resistance as well as genomic resistance profiles. When multiple publications are available, the most recent and up-to-date article has been selected and when multiple publications from the same year are present, data with the most recent sample collection or bigger sample size have been analyzed.

## MENA REGIONAL EPIDEMIOLOGY

Regarding the epidemiology of MDR P. aeruginosa, comparative regional data comprising sources of isolates, microbiological susceptibility profiles, as well as reported mechanisms of genetic resistance, including carbapenems resistance are outlined in Table 1.

**TABLE 1:** Epidemiology of MDR P. aeruginosa in the MENA region^*a*^

Country	Sample(s)	Prevalence of MDR PA^*b*^ (%)	Antimicrobial resistance^*c*^ (%)												
			PTZ	CTZ	FEP	AZT	GEN	AMK	CIP	LEV	MER	IMP	COL	Resistance genes^*d*^	Reference(s)
Iraq	General (mixed)	12.4	42.3	41.2	-	40.2	28.9	18.6	22.7	19.6	-	12.4	0	MBL: *bla*_VIM_ (33.3%), *bla*_IMP_ (25%), *bla*_NDM_ (8.3%)	65
	Urine	100% in patients without kidney disease; 88.8% in outpatients with UTI infections	-	50	-	-	38.7	27.7	38.7	38.7	-	0	-	-	66
Syria	ICU and urine	54	45.5	71.4	-	83	73	57.2	70.8	65.3	40.9	43.9	10.9	-	63^*e*^
Lebanon	General (mixed)	64.5	22	20	19	21	19	15	27	-	-	30	-	MBL: *bla*_VIM_ (16%), *bla*_IMP_ (5.71%) and *bla*_NDM_ (0%). Other mutations: *oprD* (100%); *mexXY* (68.6%), *mexCD* (34.3%), *mexAB* (31.4%), *mex*EF (0%), *ampC* (22.9%)	67–70
	ICU	33.3	28.6	28.6	28.6	42.9	28.6	28.6	28.6	-	-	42.9	-	*bla*_VIM_ (75%), *oprD* mutations (100%)	71, 72
	Urine	30	-	-	-	-	-	-	-	-	-	-	-	MBL: *bla*_VIM_ (50%), *bla*_IMP_ (16.7%) and *bla*_NDM_ (0%), *bla*_GES-6_ (75%), *bla*_KPC_ (0%); oxacillinases (0%). Other mutations: *oprD* (66.7% in CR strains), *ampC* (41.7%)	73
Palestine	General (mixed)	47.6	-	-	-	100	0	0	0	-	0	0	-	-	74
Israel	General (mixed)	30	19.6	15.7	-	-	17.6	11.8	7.8	-	17.6*		0	-	75, 76
	Urine	-	-	-	-	-	33.3*		44.4*		-	-	-	-	77
Jordan	General (mixed)	52.5	37.8	18	18	42.7	62.3	50.9	50.9	-	21.3	19.7	0	-	78
	Urine	-	-	5	-	50	15.4	0	96	12.5	-	0	-	-	79
Saudi Arabia	General (mixed)	7.3	17.2	15.5	18.9	-	16.6	5.53	18.1	-	26.3	30.7	-	*bla*_VIM_ (100%), *bla*_NDM_ (50%), *bla*_IMP_ (0%),^#^ *bla*_GES-1, 4, 6_ (8.8%), *bla*_VEB_ ESBL (47.1%), *bla*_IMP_ (29.4 %)^#^, *bla*_CTX-M_, *bla*_KPC_*, bla*_SHV,_ and *bla*_TEM_ (0% each), *bla*_OXA-10_ and *bla*_OXA-2_ (52.9%), *oprD* (43.8%), and *mexB* (43.8%) mutations	43, 56, 80–82
	ICU	61	46.3	41.8	53.3	53.1	31.7	18.8	37.5	-	52.5	38.2	30	*bla*_VIM_ (7.7%), *bla*_NDM_ (30.8%), *bla*_IMP_ (0%), *bla*_IMP,_ *bla*_KPC_ and *bla*_OXA_ (0% each). *ampC* mutations (23.1%)	44, 83
	Urine	88.9	100	100	75	-	25	25	50	-	50	-	-	-	49, 84
Kuwait	ICU	MDR rates not reported but 13.8% of all hospital- acquired infections in the neurocritical care unit are reported to be P. aeruginosa	-	-	-	-	0*		-	-	0*		-	-	85
Bahrain	General (mixed)	86% in ciprofloxacin- resistant P. aeruginosa	90	86	-	-	86	72	100	-	90	88	0	*bla*_VIM_ (50%), *bla*_NDM_ (5%), mutations: *mexXY* (4%), *mexCD* (6%), *mexAB* (4%), and *mex*EF (6%)	40, 86
Qatar	General (mixed)	8.1	90.7	-	96.6	-	73.2	58	91.2	-	90.2	-	3.4	Both class C and D β-lactamases (approxinately 96%). Dominant genes: class A β-lactamase: *bla*_VEB-9_, (25.3%), MBL: *bla*_VIM_ (24%), *bla*_IMP_ (4%) class C β-lactamase: *bla*_PDC-3_ (30.7%) and class D β-lactamase: *bla*_OXA-50_ (42.7%) and *bla*_OXA-488_ (38.7%)	51, 55
	ICU	-	7.7	15.4	12.8	-	7.7	-	7.7	-	7.7	-	0	-	87
UAE	General (mixed)	-	-	-	-	-	-	-	-	-	-	-	-	*bla*_VIM_ (32.4%), *bla*_GES-5, 9_ (5.41%), *bla*_IMP_ and *bla*_NDM_, *bla*_CTX-M_, *bla*_KPC_*, bla*_SHV_*, bla*_VEB_ and *bla*_TEM_ (0% each). Other mutations: *oprD* (73%); *mexAB* (75.6%)	58
Oman	General(mixed)	-	7	10	-	-	16	20	15	-	42	-	0	-	88
Yemen	General (mixed)	-	-	47.1	58.3	-	31.3	-	35.7	0	-	-	-	-	89
Egypt	General (mixed)	75.6 (MDR); 5.5 (pan drug resistant [resistant to all antimicrobial classes])	-	68	68	69	65	50	70	-	62	62	-	*bla*_VIM_ (52.9%), *bla*_KPC_ and *bla*_NDM_ (2.9% each), *bla*_OXA-48_ (0%). Other mutations: *oprD* (0%), *mexAB* (21.8%), *mexCD* (75%), *mexEF* (18.7%), *mexXY* (62%)	90–92
	ICU	22.5	44	-	56	-	89	44	67	44	78	78	22	*bla*_VIM_ (50%), *bla*_IMP_ (18.2%) and *bla*_NDM_ (27.3%), *bla*_KPC_ (0%), *bla*_GES_ (40.9%)	45, 93, 94
	Urine	100	36	100	74	-	6	2	8	6	-	10	-	*bla*_VEB_ and *bla*_VIM_ (0% each. Other mutations: *oprD* (100%); *mexAB* (100%), *ampC* (100%)	95
Libya	General (mixed)	-	37	66	70	37	91	79	91	-	79	87	0	*bla*_VIM-2_ (90.5%), *bla*_IMP,_ *bla*_KPC_ and *bla*_NDM_ (0% each). Other mutations: *oprD* (100%)	96
	ICU	36.4	46	55	46	64	64	36	55	55	46	36	0	*-*	97
	Urine	-	0	11.1	0	33.3	0	0	11.1	11.1	22.2	11.1	-	-	98
Sudan	General (mixed)	-	-	-	-	-	-	-	-	-	61.1*		-	-	99
Tunisia	General (mixed)	54	-	70	-	-	96	67	100	-	-	74	-	-	100
	ICU	-	0	28.4	0	52.2	0	0	0	-	-	53.7	-	*bla*_VIM-2_ (38.8%), *bla*_IMP,_ *bla*_KPC,_ *bla*_OXA-48_ and *bla*_GES_ (0% each). Other mutations: *oprD* (30.6%)	101
Algeria	General (mixed)	-	-	15	-	0	26	31	0	2	-	20.8	-	*bla*_SHV-11_ (100%), *bla*_VIM-4_ (100%), *bla*_IMP,_ *bla*_NDM,_ *bla*_CTX-M,_ *bla*_KPC,_ *bla*_OXA,_ *bla*_TEM,_ *bla*_VEB_ and *bla*_GES_ (0% each). Other mutations: *oprD* (45.5%), *ampC* (71%)	57, 102–104
Morocco	General (mixed)	0	-	5.8	-	27.1	-	0.6	11	-	14.2	7.7	-	*bla*_VIM_ (6.06%), *bla*_IMP,_ *bla*_NDM,_ *bla*_KPC,_ *bla*_OXA,_ and *bla*_GES_ (0% each)	105, 106
	ICU	28.5	-	-	-	-	-	-	-	-	-	-	-	-	107

a Of note, percentages of resistance reported by different studies as such are not directly comparable, as the studies vary in selection criteria and period of time and often employ distinct antimicrobial susceptibility methodologies and interpretative criteria.

b MDR PA rates represent the most recent reported MDR rates in P. aeruginosa in the respective country.

c The antimicrobial resistance rates are the percentages of P. aeruginosa resistant to the respective antibiotic: PTZ, piperacillin-tazobactam; CTZ, ceftazidime; FEP, cefepime; AZT, aztreonam; GEN, gentamicin; AMK, amikacin; CIP, ciprofloxacin; LEV, levofloxacin; MER, meropenem; IMP, imipenem; COL, colistin. Values that were reported as resistance rate to antibiotic groups (carbapenem/aminoglycoside/fluoroquinolone) rather than to individual antibiotics are indicated with an asterisk. -, not reported/available.

d In the Resistance genes column, the genes and the percentages of the carbapenem-resistant (CR) P. aeruginosa strains with the reported gene from these countries are shown. MBL, metallo-β-lactamase. Values that were results from different studies are indicated with a pound sign (^#^). -, not reported/available.

e The disk diffusion method was used in this study to determine colistin resistance. This methodology is not recommended by the Clinical and Laboratory Standards Institute or the European Committee on Antimicrobial Susceptibility Testing.

## COMPARATIVE REVIEW

### Prevalence of MDR P. aeruginosa.

**FIG 2:**
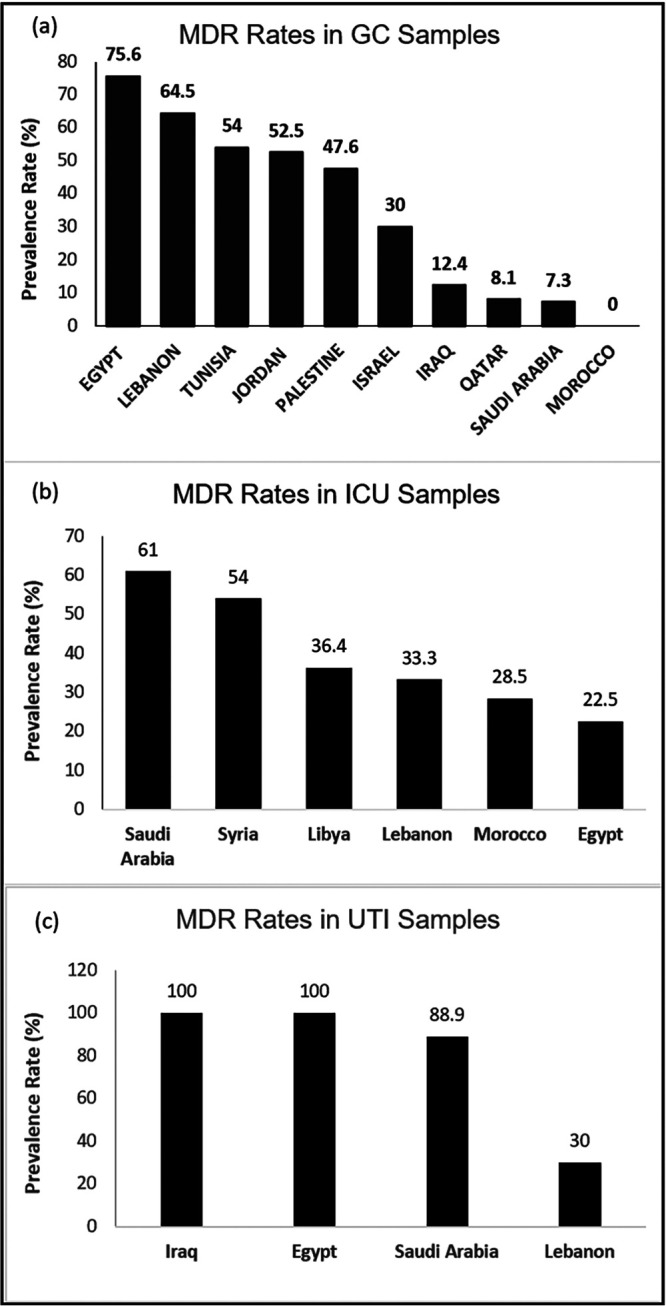
MDR P. aeruginosa prevalence rates in the MENA region. The various countries are shown on the *x* axis. The *y* axis shows the prevalence percentage of MDR P. aeruginosa among total P. aeruginosa infections in general clinical (GC) samples (a), intensive care unit (ICU) samples (b), and urinary tract infection (UTI) samples (c).

In the MENA region, there are wide regional and interregional variations in the reported prevalence of MDR P. aeruginosa from general clinical samples with the highest prevalence in Egypt (75.6%) and lowest prevalence in Morocco (0%), with modest prevalence in Saudi Arabia (7.3%) and Qatar (8.1%) (Fig. 2a). The Levant countries (Iraq, Lebanon, Palestine, Israel, and Jordan) were similarly variable with high-level resistance in Lebanon (64.5%), Jordan (52.5%), Palestine (47.6%), and Israel (30%) compared to Iraq (12.4%). This might reflect comparable regional culture of liberal antibiotic prescribing or nonuniform ASPs. It is worth mentioning that it is vital to interpret reported data cautiously to avoid selection biases since it might reflect different study methods or source locations. For example, the high rates of multidrug resistance (86%) reported from Bahrain stems from preexisting observed selection of high-level ciprofloxacin resistance (100%) from collected samples of ciprofloxacin-resistant P. aeruginosa (Table 1) (40). Of interest is the fact that reported ciprofloxacin high-level resistance is associated with concordant high-level resistance to broad-spectrum antipseudomonal agents such as piperacillin-tazobactam (90%) and carbapenems (88 to 90%), supporting observations that it is associated with other resistance mechanisms in MDR P. aeruginosa such as overproduction of efflux pumps (41). It is also interesting to note that in the Kingdom of Bahrain, carbapenem-resistant MDR P. aeruginosa isolates have been demonstrated to be mainly derived by *bla*_VIM_ as in neighboring countries but uniquely differ in harboring *bla*_NDM_, which is rare or absent in the region except for Saudi Arabia, Iraq, and Egypt (41–45). Comparative neighboring countries with similar population demographics such as Saudi Arabia and Qatar have similar prevalence of MDR P. aeruginosa (7.3% and 8.1%, respectively), which might reflect similar structured health care systems as well as antibiotic-prescribing culture compared to other GCC countries. It is needless to say that infection control and prevention together with effective antimicrobial stewardship programs (ASPs) are crucial concepts against the fight to control the spread of AMR, including P. aeruginosa (46).

While analyzing isolated samples of MDR P. aeruginosa in the region, it is important to pay attention to variations in the community or hospital settings as well as collected sample locations. The prevalence of MDR P. aeruginosa from ICU samples varied widely between the MENA region countries (Fig. 2b) with a discrepant opposite trend to that of general clinical samples. The highest rates were seen in Saudi Arabia (61%) and Syria (54%) compared to Egypt (22.5%), Libya (36.4%), Lebanon (33.3%), and Morocco (28.5%). Again, this probably reflects embedded culture of high antibiotic prescribing at critical care units or raises valid questions of efficiency of infection control and prevention measures as observed in multicenter studies from the region as well as potential possibilities of circulating or endemic high resistant clones (42, 47, 48). To emphasize the importance of sample diversity, out of the four countries for which urinary tract infection data are available, there was high-level resistance in Iraq (100%), Egypt (100%), and Saudi Arabia (88.9%,) demonstrating difficulties in managing UTIs secondary to MDR P. aeruginosa (Fig. 2c) (49). In contrast, Lebanon had a much lower rate of MDR P. aeruginosa at 30% (Fig. 2c).

While highlighting the epidemiology of MDR P. aeruginosa in the region, it is worth signifying the role of ASPs in directing appropriate and judicious prescribing of antibiotics particularly in secondary care. The concept is relatively new in many MENA countries with plans to roll it out both at primary and secondary care levels. A study from Qatar demonstrated that the introduction of an effective ASP in 2015 managed to steadily reduce the prevalence of MDR P. aeruginosa from 9% to 5.46% over a 3-year period (16).

### Antibiotic resistance patterns.

**FIG 3:**
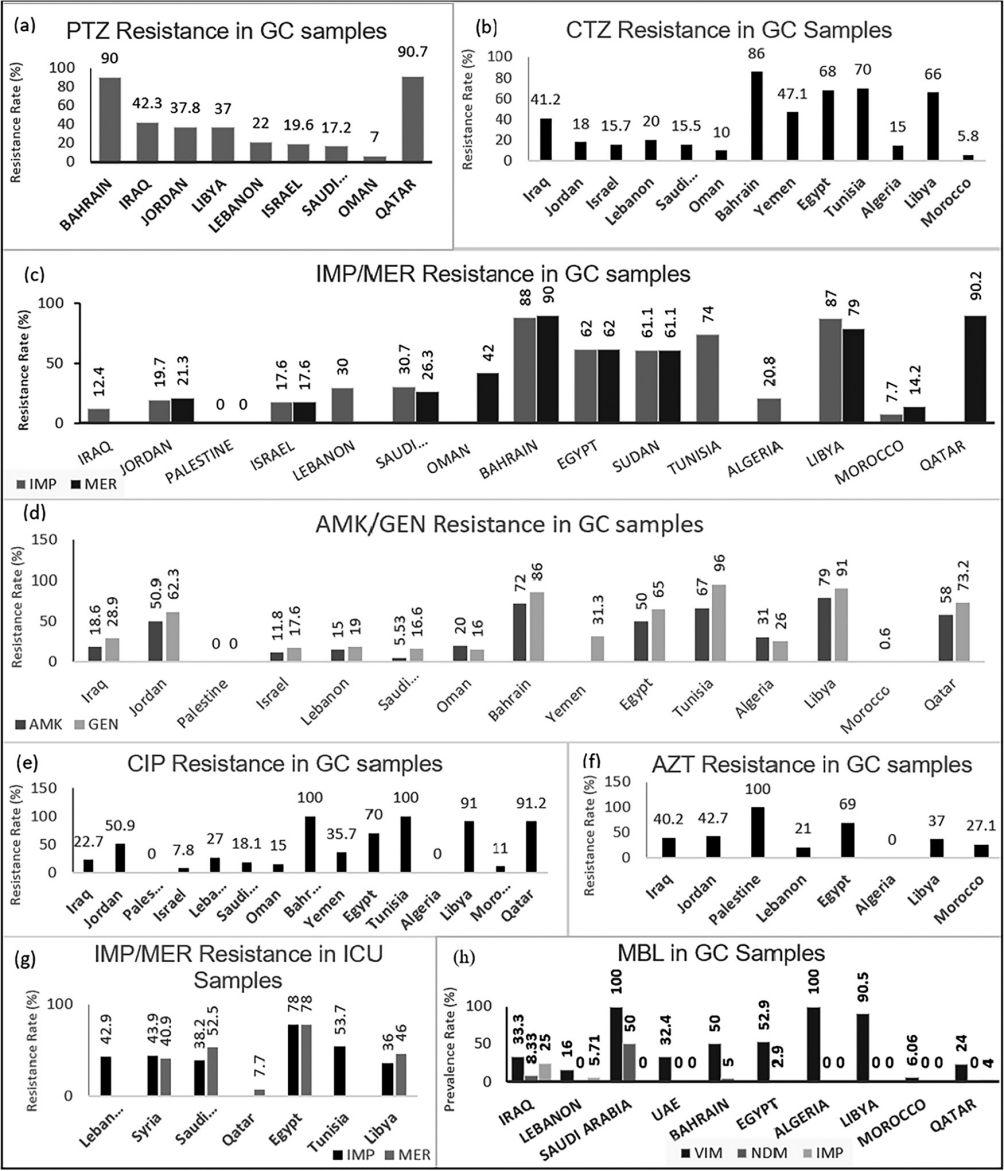
Antibiotic resistance profile of P. aeruginosa in the MENA region. The various countries are shown on the *x* axis. The *y* axis represents the resistance percentage in general clinical (GC) samples (a to f) and ICU samples (g) to piperacillin-tazobactam (PTZ) (a), ceftazidime (CTZ) (b), imipenem (IMP) and meropenem (MER) (c), amikacin (AMK) and gentamicin (GEN) (d), ciprofloxacin (CIP) (e), aztreonam (AZT) (f), and imipenem (IMP) and meropenem (MER) (g). (h) Genetic profiles of carbapenem-resistant P. aeruginosa from general clinical samples. Countries for which no value is shown have no reported data for the respective antibiotic.

When comparing general clinical samples, the overall antipseudomonal drug resistance of the ciprofloxacin-resistant Pseudomonas strains from Bahrain is high, ranging between 72 and 100% for third-generation cephalosporins, carbapenems, aminoglycosides, fluoroquinolones, and piperacillin-tazobactam combinations (Fig. 3). Resistance to piperacillin-tazobactam is moderate in Iraq (42.3%), Jordan (37.8%), Libya (37%), and Lebanon (22%), while it was low in Israel (19.6%), Saudi Arabia (17.2%), and Oman (7%) (Fig. 3a). As for antimicrobial susceptibility for third- and fourth-generation antipseudomonal cephalosporins, they are characteristically high, demonstrating low-level resistance, but exceptionally high resistance is seen in Qatar (96.6%), Bahrain (86%), Tunisia (70%), Egypt (68%), Libya (66%), Yemen (47.1%), and Iraq (41.2%) (Table 1 and Fig. 3b). In most countries, prevalent resistance rates for both ceftazidime and cefepime are similar. In Jordan, P. aeruginosa isolates showed low-level resistance to ceftazidime and cefepime (18% each) but mid-range resistance to aminoglycosides and fluoroquinolones (50.9 to 62.3% and 50.9%, respectively) (Fig. 3b, d, and e). Similar to the high prevalence of cephalosporin resistance, high carbapenem resistance has been observed in Qatar (90.2%), Bahrain (88 to 90%), Egypt (62%), Sudan (61.1%), Libya (79 to 87%), and Tunisia (74%) (Fig. 3c, d, and e) along with high-level resistance to aminoglycosides and fluoroquinolones, except for Sudan for which these data were not available. Even though P. aeruginosa reported from Saudi Arabia and Oman has low-level resistance to cephalosporin, these isolates had higher carbapenem resistance rates of 26.3 to 30.7% and 42%, respectively (Fig. 3c). Resistance to aztreonam was high in all countries where data were available, with Palestine showing absolute resistance in all tested isolates. Algeria was an exception to this with none of the isolates showing resistance to monobactams (Fig. 3f), which raises potential possibilities of interregional variations of underlying resistance mechanisms such as class A ESBL which limits aztreonam activity as opposed to class B *β*-lactamases which aztreonam is capable to overcome (50).

Worryingly, the rising trends of rates of resistance to carbapenems in most countries in the region are alarming, since it has been the forefront class to combat AMR particularly in *Enterobacteriaceae* as well as P. aeruginosa, thus limiting available treatment options. In contrast, among general clinical samples, colistin remains highly active, approaching 100% in most regions, including GCC, although developing resistance is being seen in some countries like Qatar (3.4%) (51). Novel antibiotics such as ceftazidime-avibactam and ceftolozane-tazobactam demonstrated good antimicrobial susceptibilities in Gulf countries, but it is less compared to other regions probably because of high regional resistance such as in Qatar where 31.2% and 37.1% of the MDR strains showed resistance to ceftazidime-avibactam and ceftolozane-tazobactam, respectively, even before their introduction into clinical practice (52, 53). Such observation of lower susceptibility profiles for potent novel antibiotics not previously used in the region is worrisome, since it reflects significant embedded resistance (52). Despite its wide mechanism of action against multidrug-resistant organisms such as MDR P. aeruginosa, both ceftazidime-avibactam and ceftolozane-tazobactam remain vulnerable when encountering class B MBL-producing β-lactamases (54). That might explain some of the observed lower antimicrobial susceptibilities for the drugs in the region (52).

In general, antimicrobial susceptibility data for the ICU isolates were available for fewer countries (see Fig. S1 in the supplemental material). Out of the countries that reported antimicrobial susceptibility data from ICUs, Qatar is the only country that showed low-level resistance to all tested antibiotics with rates not exceeding 15.4% for any antibiotic and absolute sensitivity to colistin (Fig. 3g and Fig. S1). Saudi Arabia, Egypt, Syria, Libya, and Lebanon consistently showed high resistance levels for piperacillin-tazobactam, cephalosporins, carbapenems, monobactams, aminoglycosides, and fluoroquinolones except Egypt where data were not available for monobactams (Fig. 3g and Fig. S1). Additionally, from available reports for Saudi Arabia, Egypt, and Syria, the prevalent isolates from ICU in these regions are also showing increasing resistance to colistin (10.9 to 30%) (Fig. S1). Despite showing very high rates of resistance to all other tested antibiotics, isolates in Libya showed absolute susceptibility to colistin (Fig. S1e). Tunisia demonstrated a little different trend with high resistance to ceftazidime and carbapenems but no resistance to piperacillin-tazobactam, ciprofloxacin, and aminoglycosides (0%) (Fig. S1 and Fig. 3g). All countries with available data showed high resistance to aztreonam (Fig. S1).

10.1128/mSphere.00202-21.1 FIG S1 Antibiotic resistance patterns of P. aeruginosa isolated from ICU samples in the MENA region. The *x* axis shows the various countries; the *y* axis shows the resistance percentage to (a) piperacillin-tazobactam (PTZ), (b) ceftazidime (CTZ)/cefepime (FEP), (c) ciprofloxacin (CIP), (d) amikacin (AMK)/gentamicin (GEN), (e) colistin (COL), and (f) aztreonam (AZT). Countries for which no value is shown have no reported data for the respective antibiotic. Download FIG S1, DOCX file, 0.1 MB. Copyright © 2021 Al-Orphaly et al. 2021 Al-Orphaly et al. https://creativecommons.org/licenses/by/4.0/ This content is distributed under the terms of the Creative Commons Attribution 4.0 International license.

Data were available for few select countries in the MENA region for UTI infections, and the available resistance patterns were very variable among UTI samples from different countries. Uropathogenic P. aeruginosa isolates from Saudi Arabia consistently showed high rates of resistance to all tested antibiotics: piperacillin-tazobactam, cephalosporins, carbapenems, fluoroquinolones, and aminoglycosides (Fig. S2). Out of three countries (Saudi Arabia, Egypt, and Libya) that reported data on piperacillin-tazobactam resistance, Saudi Arabia had the highest resistance rates (100%), followed by Egypt (36%), and Libya reported no resistance (Fig. S2a). For cephalosporins, Saudi Arabia (100%, 75%), Egypt (100%, 74%), and Iraq (50% and not available) showed highest rates of resistance to ceftazidime and cefepime, respectively (Fig. S2b). Interestingly, from countries that reported carbapenem resistance data for UTI samples, only Saudi Arabia and Libya reported high rates of resistance to meropenem (50% and 22.2%, respectively) (Fig. S2c) compared to Egypt (10%), Libya (11.1%), Iraq and Jordan (0% each), all reporting low or no resistance to imipenem (Fig. S2c). Resistance to aminoglycosides (0 to 6%) and fluoroquinolones (8 to 11.1%) was also low in both Egypt and Libya (Fig. S2d and e). Saudi Arabia, Iraq, and Israel showed high resistance to aminoglycosides (25%, 27.7 to 38.7% and 33.3%, respectively) as well as fluoroquinolones (50%, 38.7%, and 44.4%, respectively) (Fig. S2d and e).

10.1128/mSphere.00202-21.2 FIG S2 Antibiotic resistance patterns of P. aeruginosa isolated from urinary tract infection (UTI) samples in the MENA region. The *x* axis shows the various countries; the *y* axis shows the resistance percentage to (a) piperacillin-tazobactam (PTZ), (b) ceftazidime (CTZ)/cefepime (FEP), (c) imipenem (IMP)/meropenem (MER), (d) amikacin (AMK)/gentamicin (GEN), and (e) ciprofloxacin (CIP). Countries for which no value is shown have no reported data for the respective antibiotic. Download FIG S2, DOCX file, 1.0 MB. Copyright © 2021 Al-Orphaly et al. 2021 Al-Orphaly et al. https://creativecommons.org/licenses/by/4.0/ This content is distributed under the terms of the Creative Commons Attribution 4.0 International license.

### Genetic profiles.

Although genomic studies are vital in understanding the epidemiology of AMR, including MDR P. aeruginosa, genetic data on MDR *P. aeruginosa* is not widely available in the MENA region with few studies exploring the concept. The more developed countries have more available data compared to the rest particularly among highly carbapenem-resistant P. aeruginosa (Fig. 3h and Fig. S3). For example, there was little data regarding ESBL production but there was an overall predominance of *bla*_GES_ and *bla*_VEB_ *β*-lactamase genes in MDR P. aeruginosa in the region (55, 56). In the general clinical samples, the main reported ESBL genes were *bla*_GES-1,4,6_ (8.8%) and *bla*_VEB_ (47.1%) in Saudi Arabia, *bla*_GES-5,9_ (5.41%) in UAE, *bla*_VEB_ (25.3%) in Qatar, as well as *bla*_SHV_ (100%) in Algeria (Table 1) (55–58). Noteworthy, there are variations reported in *bla*_GES_ when examined through molecular/genomic testing methods (59–61). While *bla*_GES-1,7,19_ are ESBLs, some others like *bla*_GES-5,16,20,_ have carbapenemase activity and as the gene is not always sequenced in many studies, this limits the discrimination of the variant.

10.1128/mSphere.00202-21.3 FIG S3 Genetic profiles of carbapenem-resistant (CR) P. aeruginosa from general clinical (GC) and ICU samples in the MENA region. Asterisks indicate that the percentages for efflux pumps for Bahrain are from all P. aeruginosa isolates where 90% of them were carbapenem resistant. Gaps/blanks represent data not reported from these countries. The *x* axis shows the various countries; the *y* axis shows the prevalence of (a) OprD mutations, (b) efflux pumps, and (c) MBL found in carbapenem-resistant P. aeruginosa from ICU samples in the MENA region. Countries for which no value is shown have no reported data for the respective antibiotic. Download FIG S3, DOCX file, 0.04 MB. Copyright © 2021 Al-Orphaly et al. 2021 Al-Orphaly et al. https://creativecommons.org/licenses/by/4.0/ This content is distributed under the terms of the Creative Commons Attribution 4.0 International license.

In general, for clinical samples from all countries where data were available, *bla*_VIM_ is the most prevalent MBL, followed by *bla*_IMP_*/bla*_NDM_. For instance, in Iraq, *bla*_IMP_ is the second most prevalent, whereas in Saudi Arabia, *bla*_NDM_ is the second most prevalent (Fig. 3h). For P. aeruginosa isolates from ICU samples in the MENA region, extremely limited data on MBL genes were available. Among the four countries that reported MBL data and like general clinical samples from the region, *bla*_VIM_ was mostly the most prevalent MBL gene (Fig. S3c) among the MDR P. aeruginosa ICU isolates. In contrast to other regions, *bla*_NDM_ is rare in MDR P. aeruginosa ICU isolates in the MENA region, as it was identified in ICU isolates from only two countries: Saudi Arabia (30.8%) and Egypt (27.3%) (Fig. S3c) (44, 45, 47).

Mutations of the *oprD* gene were also prevalent in several countries, with the highest rates reported in Lebanon and Libya, where all carbapenem-resistant P. aeruginosa strains contained *oprD* mutations (Fig. S3a). None of the carbapenem-resistant strains in Egypt contained a mutation in the *oprD* gene (Fig. S3a). Among countries that reported MexAB efflux pump dysregulation, UAE showed the highest (75.6%) rate (Fig. S3b). MexXY efflux pump was highly prevalent in Lebanon (68.6%) and Egypt (62%) (Fig. S3b).

## CONCLUSION

The MENA region is geographically close but with diverse economic, social, and cultural differences which are reflected in health care and resources, including liberal or limited antibiotic prescribing and consumption. There is an observed diversity in the epidemiology of MDR P. aeruginosa across the region in terms of antimicrobial resistance and genetic profiles. The overall paucity of published literature on AMR in the MENA region is concerning, in conjunction with unmatched studies in terms of sampling or methodology. Nevertheless, the observed variations could also be because of differences in the structure of health systems with variable settings between high- and low-income countries, variations in population demographics or differences in antibiotic prescribing across health care sectors, or inadequate implementation of infection control and prevention measures. Understanding these differences is crucial to allow for accurate identification, followed by appropriate interventions.

This minireview also highlights the alarming situation of AMR in the MENA region with significant resistance profile for MDR P. aeruginosa limiting treatment options with all its deleterious consequences. It is imperative to obtain comprehensive collected data toward the control of AMR, which should be encouraged to limit its propagation in the MENA region. Although many countries in the region have implemented ASPs to support existing infection control practitioners, there is a clear need for regional cooperation to share challenges and experience. Last, such surveillance should be brought to the attention of policy and health care decision makers since it will have direct impact on health outcomes as well as on health care expenditure. It should also encourage creation of regional surveillance programs to monitor AMR as advocated by the WHO and leading infection bodies (62).

Despite this minireview covering an important aspect of AMR in a previously unexplored region, there are several study limitations. As highlighted, there is an absence of uniform comprehensive data and AMR reporting mechanisms from the MENA region. Also, there are no data from many countries in the region, which reflects a fundamental problem in published research from the region. Therefore, selecting individual non-national studies might certainly generate inaccurate reporting bias for prevalence rates, microbiological characteristics, or mechanisms of genetic resistance. Of note, when comparing antimicrobial susceptibility data from different studies regionally or globally, it is also important to keep in mind the differences between studies and the percentages of resistance reported by different studies because these studies are not directly comparable as they vary in selection criteria and period of time and often employ different antimicrobial susceptibility methodologies and interpretative criteria. For example, a previous study reporting colistin resistance (63) utilized disk diffusion methods which are less reliable compared to the current recommended practice of broth microdilution (64). Nevertheless, this minireview fills an important void in the literature and highlights that there are close similarities between neighboring countries, which supports that projected observations are interregionally reliable.

## References

[1] Centers for Disease Control and Prevention. 2019. Antibiotic resistance threats in the United States. Centers for Disease Control and Prevention, Atlanta, GA. www.cdc.gov/DrugResistance/Biggest-Threats.html.

[2] European Commission. 2017. EU Action on antimicrobial resistance. European Commission, Brussels, Belgium.

[3] The Review on Antimicrobial Resistance. 2016. Tackling drug-resistant infections globally: final report and recommendations. The Review on Antimicrobial Resistance, London, United Kingdom. https://amr-review.org.

[4] Bassetti M, Carnelutti A, Peghin M. 2017. Patient specific risk stratification for antimicrobial resistance and possible treatment strategies in gram-negative bacterial infections. Expert Rev Anti Infect Ther 15:55–65. doi:10.1080/14787210.2017.1251840. 27766913

[5] Zilberberg MD, Shorr AF, Micek ST, Vazquez-Guillamet C, Kollef MH. 2014. Multi-drug resistance, inappropriate initial antibiotic therapy and mortality in Gram-negative severe sepsis and septic shock: a retrospective cohort study. Crit Care 18:596. doi:10.1186/s13054-014-0596-8. 25412897 PMC4264255

[6] Bassetti M, De Waele JJ, Eggimann P, Garnacho-Montero J, Kahlmeter G, Menichetti F, Nicolau DP, Paiva JA, Tumbarello M, Welte T, Wilcox M, Zahar JR, Poulakou G. 2015. Preventive and therapeutic strategies in critically ill patients with highly resistant bacteria. Intensive Care Med 41:776–795. doi:10.1007/s00134-015-3719-z. 25792203 PMC7080151

[7] Boyle DP, Zembower TR. 2015. Epidemiology and management of emerging drug-resistant Gram-negative bacteria: extended-spectrum beta-lactamases and beyond. Urol Clin North Am 42:493–505. doi:10.1016/j.ucl.2015.05.005. 26475946

[8] Lister PD, Wolter DJ, Hanson ND. 2009. Antibacterial-resistant *Pseudomonas aeruginosa*: clinical impact and complex regulation of chromosomally encoded resistance mechanisms. Clin Microbiol Rev 22:582–610. doi:10.1128/CMR.00040-09. 19822890 PMC2772362

[9] Bassetti M, Vena A, Croxatto A, Righi E, Guery B. 2018. How to manage *Pseudomonas aeruginosa* infections. Drugs Context 7:212527. doi:10.7573/dic.212527. 29872449 PMC5978525

[10] Boucher HW, Talbot GH, Bradley JS, Edwards JE, Gilbert D, Rice LB, Scheld M, Spellberg B, Bartlett J. 2009. Bad bugs, no drugs: no ESKAPE! An update from the Infectious Diseases Society of America. Clin Infect Dis 48:1–12. doi:10.1086/595011. 19035777

[11] Tacconelli E, Carrara E, Savoldi A, Harbarth S, Mendelson M, Monnet DL, Pulcini C, Kahlmeter G, Kluytmans J, Carmeli Y, Ouellette M, Outterson K, Patel J, Cavaleri M, Cox EM, Houchens CR, Grayson ML, Hansen P, Singh N, Theuretzbacher U, Magrini N, WHO Pathogens Priority List Working Group. 2018. Discovery, research, and development of new antibiotics: the WHO priority list of antibiotic-resistant bacteria and tuberculosis. Lancet Infect Dis 18:318–327. doi:10.1016/S1473-3099(17)30753-3. 29276051

[12] Ahmed SA, Karanis P. 2020. *Cryptosporidium* and cryptosporidiosis: the perspective from the Gulf countries. Int J Environ Res Public Health 17:6824. doi:10.3390/ijerph17186824. PMC7558405 32962045

[13] Morgan DJ, Okeke IN, Laxminarayan R, Perencevich EN, Weisenberg S. 2011. Non-prescription antimicrobial use worldwide: a systematic review. Lancet Infect Dis 11:692–701. doi:10.1016/S1473-3099(11)70054-8. 21659004 PMC3543997

[14] Khalifeh MM, Moore ND, Salameh PR. 2017. Self-medication misuse in the Middle East: a systematic literature review. Pharmacol Res Perspect 5:e00323. doi:10.1002/prp2.323. PMC5684864 28805984

[15] Hashad N, Perumal D, Stewart D, Tonna AP. 2020. Mapping hospital antimicrobial stewardship programmes in the Gulf Cooperation Council states against international standards: a systematic review. J Hosp Infect 106:404–418. doi:10.1016/j.jhin.2020.09.004. 32911008

[16] Sid Ahmed MA, Abdel Hadi H, Abu Jarir S, Al Khal AL, Al-Maslamani MA, Jass J, Ibrahim EB, Ziglam H. 2020. Impact of an antimicrobial stewardship programme on antimicrobial utilization and the prevalence of MDR *Pseudomonas aeruginosa* in an acute care hospital in Qatar. JAC-Antimicrob Resist 2:dlaa050. doi:10.1093/jacamr/dlaa050. 34223010 PMC8210253

[17] Pandey N, Cascella M. 2020. Beta lactam antibiotics. StatPearls Publishing, Treasure Island, FL. https://www.ncbi.nlm.nih.gov/books/NBK545311/. 31424895

[18] Bush K. 2018. Past and present perspectives on beta-lactamases. Antimicrob Agents Chemother 62:e01076-18. doi:10.1128/AAC.01076-18. 30061284 PMC6153792

[19] Weldhagen GF, Poirel L, Nordmann P. 2003. Ambler class A extended-spectrum beta-lactamases in *Pseudomonas aeruginosa*: novel developments and clinical impact. Antimicrob Agents Chemother 47:2385–2392. doi:10.1128/AAC.47.8.2385-2392.2003. 12878494 PMC166056

[20] Horcajada JP, Montero M, Oliver A, Sorli L, Luque S, Gomez-Zorrilla S, Benito N, Grau S. 2019. Epidemiology and treatment of multidrug-resistant and extensively drug-resistant Pseudomonas aeruginosa infections. Clin Microbiol Rev 32:e00031-19. doi:10.1128/CMR.00031-19. 31462403 PMC6730496

[21] Livermore DM. 2002. Multiple mechanisms of antimicrobial resistance in *Pseudomonas aeruginosa*: our worst nightmare? Clin Infect Dis 34:634–640. doi:10.1086/338782. 11823954

[22] Cabot G, Ocampo-Sosa AA, Tubau F, Macia MD, Rodriguez C, Moya B, Zamorano L, Suarez C, Pena C, Martinez-Martinez L, Oliver A, Spanish Network for Research in Infectious Diseases (REIPI). 2011. Overexpression of AmpC and efflux pumps in *Pseudomonas aeruginosa* isolates from bloodstream infections: prevalence and impact on resistance in a Spanish multicenter study. Antimicrob Agents Chemother 55:1906–1911. doi:10.1128/AAC.01645-10. 21357294 PMC3088238

[23] Juan C, Torrens G, Gonzalez-Nicolau M, Oliver A. 2017. Diversity and regulation of intrinsic beta-lactamases from non-fermenting and other Gram-negative opportunistic pathogens. FEMS Microbiol Rev 41:781–815. doi:10.1093/femsre/fux043. 29029112

[24] Moya B, Dotsch A, Juan C, Blazquez J, Zamorano L, Haussler S, Oliver A. 2009. Beta-lactam resistance response triggered by inactivation of a nonessential penicillin-binding protein. PLoS Pathog 5:e1000353. doi:10.1371/journal.ppat.1000353. 19325877 PMC2654508

[25] Cabot G, Bruchmann S, Mulet X, Zamorano L, Moya B, Juan C, Haussler S, Oliver A. 2014. *Pseudomonas aeruginosa* ceftolozane-tazobactam resistance development requires multiple mutations leading to overexpression and structural modification of AmpC. Antimicrob Agents Chemother 58:3091–3099. doi:10.1128/AAC.02462-13. 24637685 PMC4068469

[26] Fraile-Ribot PA, Cabot G, Mulet X, Perianez L, Martin-Pena ML, Juan C, Perez JL, Oliver A. 2018. Mechanisms leading to in vivo ceftolozane/tazobactam resistance development during the treatment of infections caused by MDR *Pseudomonas aeruginosa*. J Antimicrob Chemother 73:658–663. doi:10.1093/jac/dkx424. 29149337

[27] Kohler T, Epp SF, Curty LK, Pechere JC. 1999. Characterization of MexT, the regulator of the MexE-MexF-OprN multidrug efflux system of *Pseudomonas aeruginosa*. J Bacteriol 181:6300–6305. doi:10.1128/JB.181.20.6300-6305.1999. 10515918 PMC103763

[28] Lopez-Causape C, Sommer LM, Cabot G, Rubio R, Ocampo-Sosa AA, Johansen HK, Figuerola J, Canton R, Kidd TJ, Molin S, Oliver A. 2017. Evolution of the *Pseudomonas aeruginosa* mutational resistome in an international cystic fibrosis clone. Sci Rep 7:5555. doi:10.1038/s41598-017-05621-5. 28717172 PMC5514035

[29] Riera E, Cabot G, Mulet X, Garcia-Castillo M, del Campo R, Juan C, Canton R, Oliver A. 2011. *Pseudomonas aeruginosa* carbapenem resistance mechanisms in Spain: impact on the activity of imipenem, meropenem and doripenem. J Antimicrob Chemother 66:2022–2027. doi:10.1093/jac/dkr232. 21653605

[30] Moya B, Beceiro A, Cabot G, Juan C, Zamorano L, Alberti S, Oliver A. 2012. Pan-beta-lactam resistance development in *Pseudomonas aeruginosa* clinical strains: molecular mechanisms, penicillin-binding protein profiles, and binding affinities. Antimicrob Agents Chemother 56:4771–4778. doi:10.1128/AAC.00680-12. 22733064 PMC3421878

[31] Bruchmann S, Dotsch A, Nouri B, Chaberny IF, Haussler S. 2013. Quantitative contributions of target alteration and decreased drug accumulation to *Pseudomonas aeruginosa* fluoroquinolone resistance. Antimicrob Agents Chemother 57:1361–1368. doi:10.1128/AAC.01581-12. 23274661 PMC3591863

[32] Lopez-Causape C, Cabot G, Del Barrio-Tofino E, Oliver A. 2018. The versatile mutational resistome of *Pseudomonas aeruginosa*. Front Microbiol 9:685. doi:10.3389/fmicb.2018.00685. 29681898 PMC5897538

[33] Muller C, Plesiat P, Jeannot K. 2011. A two-component regulatory system interconnects resistance to polymyxins, aminoglycosides, fluoroquinolones, and beta-lactams in *Pseudomonas aeruginosa*. Antimicrob Agents Chemother 55:1211–1221. doi:10.1128/AAC.01252-10. 21149619 PMC3067119

[34] Botelho J, Grosso F, Peixe L. 2018. Unravelling the genome of a *Pseudomonas aeruginosa* isolate belonging to the high-risk clone ST235 reveals an integrative conjugative element housing a blaGES-6 carbapenemase. J Antimicrob Chemother 73:77–83. doi:10.1093/jac/dkx337. 29029083

[35] van der Zee A, Kraak WB, Burggraaf A, Goessens WHF, Pirovano W, Ossewaarde JM, Tommassen J. 2018. Spread of carbapenem resistance by transposition and conjugation among *Pseudomonas aeruginosa*. Front Microbiol 9:2057. doi:10.3389/fmicb.2018.02057. 30233535 PMC6133989

[36] Martis N, Leroy S, Blanc V. 2014. Colistin in multi-drug resistant *Pseudomonas aeruginosa* blood-stream infections: a narrative review for the clinician. J Infect 69:1–12. doi:10.1016/j.jinf.2014.03.001. 24631777

[37] Poole K. 2011. *Pseudomonas aeruginosa*: resistance to the max. Front Microbiol 2:65. doi:10.3389/fmicb.2011.00065. 21747788 PMC3128976

[38] Del Barrio-Tofino E, Zamorano L, Cortes-Lara S, Lopez-Causape C, Sanchez-Diener I, Cabot G, Bou G, Martinez-Martinez L, Oliver A, GEMARA-SEIMC/REIPI Pseudomonas study Group. 2019. Spanish nationwide survey on *Pseudomonas aeruginosa* antimicrobial resistance mechanisms and epidemiology. J Antimicrob Chemother 74:1825–1835. doi:10.1093/jac/dkz147. 30989186

[39] Magiorakos AP, Srinivasan A, Carey RB, Carmeli Y, Falagas ME, Giske CG, Harbarth S, Hindler JF, Kahlmeter G, Olsson-Liljequist B, Paterson DL, Rice LB, Stelling J, Struelens MJ, Vatopoulos A, Weber JT, Monnet DL. 2012. Multidrug-resistant, extensively drug-resistant and pandrug-resistant bacteria: an international expert proposal for interim standard definitions for acquired resistance. Clin Microbiol Infect 18:268–281. doi:10.1111/j.1469-0691.2011.03570.x. 21793988

[40] Al Rashed N, Joji RM, Saeed NK, Bindayna KM. 2020. Detection of overexpression of efflux pump expression in fluoroquinolone-resistant *Pseudomonas aeruginosa* isolates. Int J Appl Basic Med Res 10:37–42. doi:10.4103/ijabmr.IJABMR_90_19. 32002384 PMC6967346

[41] Rehman A, Patrick WM, Lamont IL. 2019. Mechanisms of ciprofloxacin resistance in *Pseudomonas aeruginosa*: new approaches to an old problem. J Med Microbiol 68:1–10. doi:10.1099/jmm.0.000873. 30605076

[42] Zowawi HM, Syrmis MW, Kidd TJ, Balkhy HH, Walsh TR, Al Johani SM, Al Jindan RY, Alfaresi M, Ibrahim E, Al-Jardani A, Al Salman J, Dashti AA, Sidjabat HE, Baz O, Trembizki E, Whiley DM, Paterson DL. 2018. Identification of carbapenem-resistant *Pseudomonas aeruginosa* in selected hospitals of the Gulf Cooperation Council States: dominance of high-risk clones in the region. J Med Microbiol 67:846–853. doi:10.1099/jmm.0.000730. 29664716

[43] Shaaban M, Al-Qahtani A, Al-Ahdal M, Barwa R. 2018. Molecular characterization of resistance mechanisms in *Pseudomonas aeruginosa* isolates resistant to carbapenems. J Infect Dev Ctries 11:935–943. doi:10.3855/jidc.9501. 31626599

[44] Abdalhamid B, Elhadi N, Alabdulqader N, Alsamman K, Aljindan R. 2016. Rates of gastrointestinal tract colonization of carbapenem-resistant *Enterobacteriaceae* and *Pseudomonas aeruginosa* in hospitals in Saudi Arabia. New Microbes New Infect 10:77–83. doi:10.1016/j.nmni.2016.01.014. 26933499 PMC4765740

[45] Ramadan RA, Gebriel MG, Kadry HM, Mosallem A. 2018. Carbapenem-resistant *Acinetobacter baumannii* and *Pseudomonas aeruginosa*: characterization of carbapenemase genes and E-test evaluation of colistin-based combinations. Infect Drug Resist 11:1261–1269. doi:10.2147/IDR.S170233. 30197524 PMC6112795

[46] Tacconelli E, Sifakis F, Harbarth S, Schrijver R, van Mourik M, Voss A, Sharland M, Rajendran NB, Rodriguez-Bano J, EPI-Net COMBACTE-MAGNET Group. 2018. Surveillance for control of antimicrobial resistance. Lancet Infect Dis 18:e99–e106. doi:10.1016/S1473-3099(17)30485-1. 29102325

[47] Zowawi HM, Balkhy HH, Walsh TR, Paterson DL. 2013. β-Lactamase production in key Gram-negative pathogen isolates from the Arabian Peninsula. Clin Microbiol Rev 26:361–380. doi:10.1128/CMR.00096-12. 23824364 PMC3719487

[48] Rosenthal VD, Belkebir S, Zand F, Afeef M, Tanzi VL, Al-Abdely HM, El-Kholy A, Aziz AlKhawaja SA, Demiroz AP, Sayed AF, Elahi N, Gamar-Elanbya MO, Abidi K, Ben-Jaballah N, Salama MF, Helali NJ, Abdel-Halim MM, Demaisip NL, Ahmed H, Diab HH, Molano AM, Sawan FA, Kelany A, Altowerqi R, Rushdi H, Alkamaly MA, Bohlega E, Aldossary HA, Abdelhady KM, Ikram A, Madco M, Caminade Y, Alazmi M, Mahfouz T, Abdelaziz-Yousef RH, Ibrahim A, Elawady B, Asad T, Shyrine L, Leblebicioglu H. 2020. Six-year multicenter study on short-term peripheral venous catheters-related bloodstream infection rates in 246 intensive units of 83 hospitals in 52 cities of 14 countries of Middle East: Bahrain, Egypt, Iran, Jordan, Kingdom of Saudi Arabia, Kuwait, Lebanon, Morocco, Pakistan, Palestine, Sudan, Tunisia, Turkey, and United Arab Emirates-International Nosocomial Infection Control Consortium (INICC) findings. J Infect Public Health 13:1134–1141. doi:10.1016/j.jiph.2020.03.012. 32295756

[49] Al Wutayd O, Al Nafeesah A, Adam I, Babikir I. 2018. The antibiotic susceptibility patterns of uropathogens isolated in Qassim, Saudi Arabia. J Infect Dev Ctries 12:946–952. doi:10.3855/jidc.10553. 32012123

[50] Ramsey C, MacGowan AP. 2016. A review of the pharmacokinetics and pharmacodynamics of aztreonam. J Antimicrob Chemother 71:2704–2712. doi:10.1093/jac/dkw231. 27334663

[51] Sid Ahmed MA, Hassan AAI, Abu Jarir S, Abdel Hadi H, Bansal D, Abdul Wahab A, Muneer M, Mohamed SF, Zahraldin K, Hamid JM, Alyazidi MA, Mohamed M, Sultan AA, Söderquist B, Ibrahim EB, Jass J. 2019. Emergence of multidrug- and pandrug-resistant *Pseudomonas aeruginosa* from five hospitals in Qatar. Infect Prevent Practice 1:100027. doi:10.1016/j.infpip.2019.100027. PMC8336314 34368684

[52] Sid Ahmed MA, Abdel Hadi H, Hassan AAI, Abu Jarir S, Al-Maslamani MA, Eltai NO, Dousa KM, Hujer AM, Sultan AA, Soderquist B, Bonomo RA, Ibrahim EB, Jass J, Omrani AS. 2019. Evaluation of in vitro activity of ceftazidime/avibactam and ceftolozane/tazobactam against MDR *Pseudomonas aeruginosa* isolates from Qatar. J Antimicrob Chemother 74:3497–3504. doi:10.1093/jac/dkz379. 31504587 PMC6857196

[53] Alatoom A, Elsayed H, Lawlor K, AbdelWareth L, El-Lababidi R, Cardona L, Mooty M, Bonilla MF, Nusair A, Mirza I. 2017. Comparison of antimicrobial activity between ceftolozane-tazobactam and ceftazidime-avibactam against multidrug-resistant isolates of *Escherichia coli*, *Klebsiella pneumoniae*, and *Pseudomonas aeruginosa*. Int J Infect Dis 62:39–43. doi:10.1016/j.ijid.2017.06.007. 28610832

[54] Yahav D, Giske CG, Gramatniece A, Abodakpi H, Tam VH, Leibovici L. 2020. New beta-lactam-beta-lactamase inhibitor combinations. Clin Microbiol Rev 34:e00115-20. doi:10.1128/CMR.00115-20. PMC7667665 33177185

[55] Sid Ahmed MA, Khan FA, Sultan AA, Soderquist B, Ibrahim EB, Jass J, Omrani AS. 2020. Beta-lactamase-mediated resistance in MDR-*Pseudomonas aeruginosa* from Qatar. Antimicrob Resist Infect Control 9:170. doi:10.1186/s13756-020-00838-y. 33131487 PMC7603671

[56] Al-Agamy MH, Jeannot K, El-Mahdy TS, Samaha HA, Shibl AM, Plesiat P, Courvalin P. 2016. Diversity of molecular mechanisms conferring carbapenem resistance to *Pseudomonas aeruginosa* isolates from Saudi Arabia. Can J Infect Dis Med Microbiol 2016:4379686. doi:10.1155/2016/4379686. 27597874 PMC4997076

[57] Bourafa N, Chaalal W, Bakour S, Lalaoui R, Boutefnouchet N, Diene SM, Rolain JM. 2018. Molecular characterization of carbapenem-resistant Gram-negative bacilli clinical isolates in Algeria. Infect Drug Resist 11:735–742. doi:10.2147/IDR.S150005. 29844691 PMC5961646

[58] Ayoub Moubareck C, Hammoudi Halat D, Akkawi C, Nabi A, AlSharhan MA, AlDeesi ZO, Peters CC, Celiloglu H, Karam Sarkis D. 2019. Role of outer membrane permeability, efflux mechanism, and carbapenemases in carbapenem-nonsusceptible *Pseudomonas aeruginosa* from Dubai hospitals: results of the first cross-sectional survey. Int J Infect Dis 84:143–150. doi:10.1016/j.ijid.2019.04.027. 31204002

[59] Hong JS, Choi N, Kim SJ, Choi KH, Roh KH, Lee S. 2020. Molecular characteristics of GES-type carbapenemase-producing *Pseudomonas aeruginosa* clinical isolates from long-term care facilities and general hospitals in South Korea. Microb Drug Resist 26:605–610. doi:10.1089/mdr.2019.0302. 31800356

[60] Hishinuma T, Tada T, Kuwahara-Arai K, Yamamoto N, Shimojima M, Kirikae T. 2018. Spread of GES-5 carbapenemase-producing *Pseudomonas aeruginosa* clinical isolates in Japan due to clonal expansion of ST235. PLoS One 13:e0207134. doi:10.1371/journal.pone.0207134. 30452435 PMC6242314

[61] Naas T, Poirel L, Nordmann P. 2008. Minor extended-spectrum beta-lactamases. Clin Microbiol Infect 14(Suppl 1):42–52. doi:10.1111/j.1469-0691.2007.01861.x. 18154527

[62] World Health Organization. 2015. Global action plan on antimicrobial resistance. World Health Organization, Geneva, Switzerland.

[63] Mahfoud M, Al Najjar M, Hamzeh AR. 2015. Multidrug resistance in *Pseudomonas aeruginosa* isolated from nosocomial respiratory and urinary infections in Aleppo, Syria. J Infect Dev Ctries 9:210–213. doi:10.3855/jidc.5643. 25699497

[64] Matuschek E, Ahman J, Webster C, Kahlmeter G. 2018. Antimicrobial susceptibility testing of colistin − evaluation of seven commercial MIC products against standard broth microdilution for *Escherichia coli*, *Klebsiella pneumoniae*, *Pseudomonas aeruginosa*, and *Acinetobacter* spp. Clin Microbiol Infect 24:865–870. doi:10.1016/j.cmi.2017.11.020. 29221995

[65] Al-Khudhairy MK, Al-Shammari MMM. 2020. Prevalence of metallo-beta-lactamase-producing *Pseudomonas aeruginosa* isolated from diabetic foot infections in Iraq. New Microbes New Infect 35:100661. doi:10.1016/j.nmni.2020.100661. 32194966 PMC7076140

[66] Majeed HT, Aljanaby AAJ. 2019. Antibiotic susceptibility patterns and prevalence of some extended spectrum beta-lactamases genes in Gram-negative bacteria isolated from patients infected with urinary tract infections in Al-Najaf City, Iraq. Avicenna J Med Biotechnol 11:192–201. 31057723 PMC6490404

[67] Matta R, Hallit S, Hallit R, Bawab W, Rogues AM, Salameh P. 2018. Epidemiology and microbiological profile comparison between community and hospital acquired infections: a multicenter retrospective study in Lebanon. J Infect Public Health 11:405–411. doi:10.1016/j.jiph.2017.09.005. 28970096

[68] Moghnieh R, Araj GF, Awad L, Daoud Z, Mokhbat JE, Jisr T, Abdallah D, Azar N, Irani-Hakimeh N, Balkis MM, Youssef M, Karayakoupoglou G, Hamze M, Matar M, Atoui R, Abboud E, Feghali R, Yared N, Husni R. 2019. A compilation of antimicrobial susceptibility data from a network of 13 Lebanese hospitals reflecting the national situation during 2015–2016. Antimicrob Resist Infect Control 8:41. doi:10.1186/s13756-019-0487-5. 30828445 PMC6381724

[69] Al Bayssari C, Diene SM, Loucif L, Gupta SK, Dabboussi F, Mallat H, Hamze M, Rolain JM. 2014. Emergence of VIM-2 and IMP-15 carbapenemases and inactivation of oprD gene in carbapenem-resistant *Pseudomonas aeruginosa* clinical isolates from Lebanon. Antimicrob Agents Chemother 58:4966–4970. doi:10.1128/AAC.02523-13. 24913164 PMC4136035

[70] Hammoudi Halat D, Moubareck CA, Sarkis DK. 2017. Heterogeneity of carbapenem resistance mechanisms among Gram-negative pathogens in Lebanon: results of the first cross-sectional countrywide study. Microb Drug Resist 23:733–743. doi:10.1089/mdr.2016.0077. 28080212

[71] Ismail A, El-Hage-Sleiman AK, Majdalani M, Hanna-Wakim R, Kanj S, Sharara-Chami R. 2016. Device-associated infections in the pediatric intensive care unit at the American University of Beirut Medical Center. J Infect Dev Ctries 10:554–562. doi:10.3855/jidc.7303. 27367002

[72] Nawfal Dagher T, Al-Bayssari C, Diene SM, Azar E, Rolain JM. 2019. Emergence of plasmid-encoded VIM-2-producing *Pseudomonas aeruginosa* isolated from clinical samples in Lebanon. New Microbes New Infect 29:100521. doi:10.1016/j.nmni.2019.100521. 30976429 PMC6438892

[73] Yaghi J, Fattouh N, Akkawi C, El Chamy L, Maroun RG, Khalil G. 2020. Unusually high prevalence of cosecretion of Ambler class A and B carbapenemases and nonenzymatic mechanisms in multidrug-resistant clinical isolates of *Pseudomonas aeruginosa* in Lebanon. Microb Drug Resist 26:150–159. doi:10.1089/mdr.2019.0040. 31424353

[74] Rida RH, Al Laham NA, Elmanama AA. 2018. Carbapenem resistance among clinical and environmental Gram-negative isolates recovered from hospitals in Gaza strip, Palestine. Germs 8:147–154. doi:10.18683/germs.2018.1142. 30250834 PMC6141226

[75] Glikson E, Sagiv D, Wolf M, Shapira Y. 2017. Necrotizing otitis externa: diagnosis, treatment, and outcome in a case series. Diagn Microbiol Infect Dis 87:74–78. doi:10.1016/j.diagmicrobio.2016.10.017. 27806892

[76] Averbuch D, Avaky C, Harit M, Stepensky P, Fried I, Ben-Ami T, Temper V, Peled Y, Troen H, Masarwa R, Abu Ahmad W, Weintraub M, Revel-Vilk S, Engelhard D. 2017. Non-fermentative Gram-negative rods bacteremia in children with cancer: a 14-year single-center experience. Infection 45:327–334. doi:10.1007/s15010-017-0988-1. 28205160

[77] Elnasasra A, Alnsasra H, Smolyakov R, Riesenberg K, Nesher L. 2017. Ethnic diversity and increasing resistance patterns of hospitalized community-acquired urinary tract infections in southern Israel: a prospective study. Isr Med Assoc J 19:538–542. 28971635

[78] Al Dawodeyah HY, Obeidat N, Abu-Qatouseh LF, Shehabi AA. 2018. Antimicrobial resistance and putative virulence genes of *Pseudomonas aeruginosa* isolates from patients with respiratory tract infection. Germs 8:31–40. doi:10.18683/germs.2018.1130. 29564246 PMC5845973

[79] Al Demour S, Ababneh MA. 2018. Evaluation of behavioral and susceptibility patterns in premenopausal women with recurrent urinary tract infections: a case control study. Urol Int 100:31–36. doi:10.1159/000485568. 29241191

[80] El-Saed A, Balkhy HH, Alshamrani MM, Aljohani S, Alsaedi A, Al Nasser W, El Gammal A, Almohrij SA, Alyousef Z, Almunif S, Alzahrani M. 2020. High contribution and impact of resistant gram negative pathogens causing surgical site infections at a multi-hospital healthcare system in Saudi Arabia, 2007–2016. BMC Infect Dis 20:275. doi:10.1186/s12879-020-4939-6. 32264843 PMC7140359

[81] Al-Tawfiq JA, Rabaan AA, Saunar JV, Bazzi AM. 2020. Antimicrobial resistance of gram-negative bacteria: a six-year longitudinal study in a hospital in Saudi Arabia. J Infect Public Health 13:737–745. doi:10.1016/j.jiph.2020.01.004. 32008927

[82] Memish ZA, Assiri A, Almasri M, Roshdy H, Hathout H, Kaase M, Gatermann SG, Yezli S. 2015. Molecular characterization of carbapenemase production among gram-negative bacteria in Saudi Arabia. Microb Drug Resist 21:307–314. doi:10.1089/mdr.2014.0121. 25569024

[83] Ibrahim ME. 2018. High antimicrobial resistant rates among Gram-negative pathogens in intensive care units. A retrospective study at a tertiary care hospital in southwest Saudi Arabia. Saudi Med J 39:1035–1043. doi:10.15537/smj.2018.10.22944. 30284588 PMC6201019

[84] Ahmed SS, Shariq A, Alsalloom AA, Babikir IH, Alhomoud BN. 2019. Uropathogens and their antimicrobial resistance patterns: relationship with urinary tract infections. Int J Health Sci (Qassim) 13:48–55. PMC6436442 30983946

[85] Abulhasan YB, Abdullah AA, Shetty SA, Ramadan MA, Yousef W, Mokaddas EM. 2020. Health care-associated infections in a neurocritical care unit of a developing country. Neurocrit Care 32:836–846. doi:10.1007/s12028-019-00856-8. 31562598

[86] Joji RM, Al-Rashed N, Saeed NK, Bindayna KM. 2019. Detection of VIM and NDM-1 metallo-beta-lactamase genes in carbapenem-resistant *Pseudomonas aeruginosa* clinical strains in Bahrain. J Lab Physicians 11:138–143. doi:10.4103/JLP.JLP_118_18. 31160853 PMC6543932

[87] Ali HS, Khan FY, George S, Shaikh N, Al-Ajmi J. 2016. Epidemiology and outcome of ventilator-associated pneumonia in a heterogeneous ICU population in Qatar. Biomed Res Int 2016:8231787. doi:10.1155/2016/8231787. 27382571 PMC4921639

[88] Al Rahmany D, Albeloushi A, Alreesi I, Alzaabi A, Alreesi M, Pontiggia L, Ghazi IM. 2019. Exploring bacterial resistance in Northern Oman, a foundation for implementing evidence-based antimicrobial stewardship program. Int J Infect Dis 83:77–82. doi:10.1016/j.ijid.2019.04.004. 30959249

[89] Badulla WFS, Alshakka M, Mohamed IMI. 2020. Antimicrobial resistance profiles for different isolates in Aden, Yemen: a cross-sectional study in a resource-poor setting. Biomed Res Int 2020:1810290. doi:10.1155/2020/1810290. 32382529 PMC7195635

[90] Kishk RM, Abdalla MO, Hashish AA, Nemr NA, El Nahhas N, Alkahtani S, Abdel-Daim MM, Kishk SM. 2020. Efflux MexAB-mediated resistance in *P. aeruginosa* isolated from patients with healthcare associated infections. Pathogens 9:471. doi:10.3390/pathogens9060471. PMC7350317 32549303

[91] El-Mahdy R, El-Kannishy G. 2019. Virulence factors of carbapenem-resistant *Pseudomonas aeruginosa* in hospital-acquired infections in Mansoura, Egypt. Infect Drug Resist 12:3455–3461. doi:10.2147/IDR.S222329. 31819540 PMC6844229

[92] Hassuna NA, Darwish MK, Sayed M, Ibrahem RA. 2020. Molecular epidemiology and mechanisms of high-level resistance to meropenem and imipenem in *Pseudomonas aeruginosa*. Infect Dis Resist 13:285–293. doi:10.2147/IDR.S233808. PMC6996622 32099420

[93] Hassuna NA, Mandour SA, Mohamed ES. 2020. Virulence constitution of multi-drug-resistant *Pseudomonas aeruginosa* in Upper Egypt. Infect Drug Resist 13:587–595. doi:10.2147/IDR.S233694. 32110069 PMC7036984

[94] El-Nawawy A, Ramadan MA, Antonios MA, Arafa SA, Hamza E. 2019. Bacteriologic profile and susceptibility pattern of mechanically ventilated paediatric patients with pneumonia. J Glob Antimicrob Resist 18:88–94. doi:10.1016/j.jgar.2019.01.028. 30710648

[95] Abbas HA, El-Ganiny AM, Kamel HA. 2018. Phenotypic and genotypic detection of antibiotic resistance of *Pseudomonas aeruginosa* isolated from urinary tract infections. Afr Health Sci 18:11–21. doi:10.4314/ahs.v18i1.3. 29977252 PMC6016981

[96] Mathlouthi N, Areig Z, Al Bayssari C, Bakour S, Ali El Salabi A, Ben Gwierif S, Zorgani AA, Ben Slama K, Chouchani C, Rolain JM. 2015. Emergence of carbapenem-resistant *Pseudomonas aeruginosa* and *Acinetobacter baumannii* clinical isolates collected from some Libyan hospitals. Microb Drug Resist 21:335–341. doi:10.1089/mdr.2014.0235. 25587875

[97] Zorgani A, Abofayed A, Glia A, Albarbar A, Hanish S. 2015. Prevalence of device-associated nosocomial infections caused by Gram-negative bacteria in a trauma intensive care unit in Libya. Oman Med J 30:270–275. doi:10.5001/omj.2015.54. 26366261 PMC4561650

[98] Mohammed MA, Alnour TM, Shakurfo OM, Aburass MM. 2016. Prevalence and antimicrobial resistance pattern of bacterial strains isolated from patients with urinary tract infection in Messalata Central Hospital, Libya. Asian Pac J Trop Med 9:771–776. doi:10.1016/j.apjtm.2016.06.011. 27569886

[99] Adam MA, Elhag WI. 2018. Prevalence of metallo-beta-lactamase acquired genes among carbapenems susceptible and resistant Gram-negative clinical isolates using multiplex PCR, Khartoum hospitals, Khartoum Sudan. BMC Infect Dis 18:668. doi:10.1186/s12879-018-3581-z. 30558551 PMC6296134

[100] Ben Nejma M, Sioud O, Mastouri M. 2018. Quinolone-resistant clinical strains of *Pseudomonas aeruginosa* isolated from University Hospital in Tunisia. 3 Biotech 8:1. doi:10.1007/s13205-017-1019-8. PMC5688038 29201587

[101] Chairat S, Ben Yahia H, Rojo-Bezares B, Saenz Y, Torres C, Ben Slama K. 2019. High prevalence of imipenem-resistant and metallo-beta-lactamase-producing *Pseudomonas aeruginosa* in the Burns Hospital in Tunisia: detection of a novel class 1 integron. J Chemother 31:120–126. doi:10.1080/1120009X.2019.1582168. 30849001

[102] Merradi M, Kassah-Laouar A, Ayachi A, Heleili N, Menasria T, Hocquet D, Cholley P, Sauget M. 2019. Occurrence of VIM-4 metallo-beta-lactamase-producing *Pseudomonas aeruginosa* in an Algerian hospital. J Infect Dev Ctries 13:284–290. doi:10.3855/jidc.10679. 32045372

[103] Meradji S, Barguigua A, Bentakouk MC, Nayme K, Zerouali K, Mazouz D, Chettibi H, Timinouni M. 2016. Epidemiology and virulence of VIM-4 metallo-beta-lactamase-producing *Pseudomonas aeruginosa* isolated from burn patients in eastern Algeria. Burns 42:906–918. doi:10.1016/j.burns.2016.02.023. 27156788

[104] Zaidi FZ, Dali-Yahia R, Zenati K, Yazi L, Lounes M, Aberkane S, Jean PH, Barraud O, Godreuil S, Touati A. 2019. Characterization of VIM-4 producing clinical *Pseudomonas aeruginosa* isolates from Western Algeria: sequence type and class 1 integron description. Microb Drug Resist 26:1437−1441. doi:10.1089/mdr.2019.0225. 31829797

[105] Elmouaden C, Laglaoui A, Ennanei L, Bakkali M, Abid M. 2019. Virulence genes and antibiotic resistance of *Pseudomonas aeruginosa* isolated from patients in the Northwestern of Morocco. J Infect Dev Ctries 13:892–898. doi:10.3855/jidc.10675. 32084019

[106] Maroui I, Barguigua A, Aboulkacem A, Ouarrak K, Sbiti M, Louzi H, Timinouni M, Belhaj A. 2016. First report of VIM-2 metallo-beta-lactamases producing *Pseudomonas aeruginosa* isolates in Morocco. J Infect Chemother 22:127–132. doi:10.1016/j.jiac.2015.11.008. 26711231

[107] El Mekes A, Zahlane K, Ait Said L, Tadlaoui Ouafi A, Barakate M. 2020. The clinical and epidemiological risk factors of infections due to multi-drug resistant bacteria in an adult intensive care unit of University Hospital Center in Marrakesh-Morocco. J Infect Public Health 13:637–643. doi:10.1016/j.jiph.2019.08.012. 31537511

