# Supplementary figures and images for: Epidemiology of Multidrug-Resistant Pseudomonas aeruginosa in the Middle East and North Africa Region
Source: mSphere. 2021 May 19;6(3):e00202-21. doi: 10.1128/mSphere.00202-21 (PMC8265635; doi:10.1128/mSphere.00202-21)

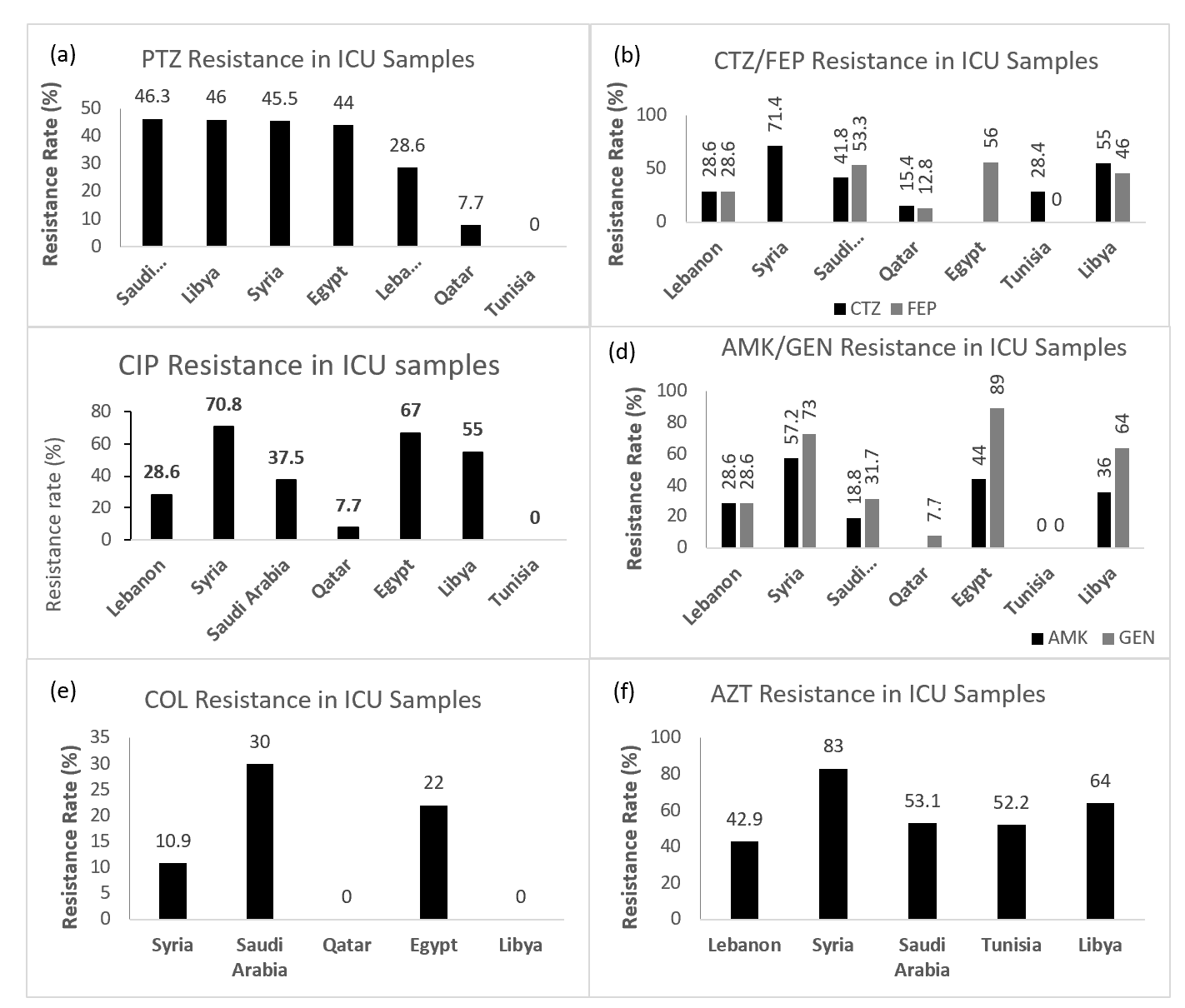


(c)

Supplement: FIG S1 [file msphere.00202-21-sf001.docx]

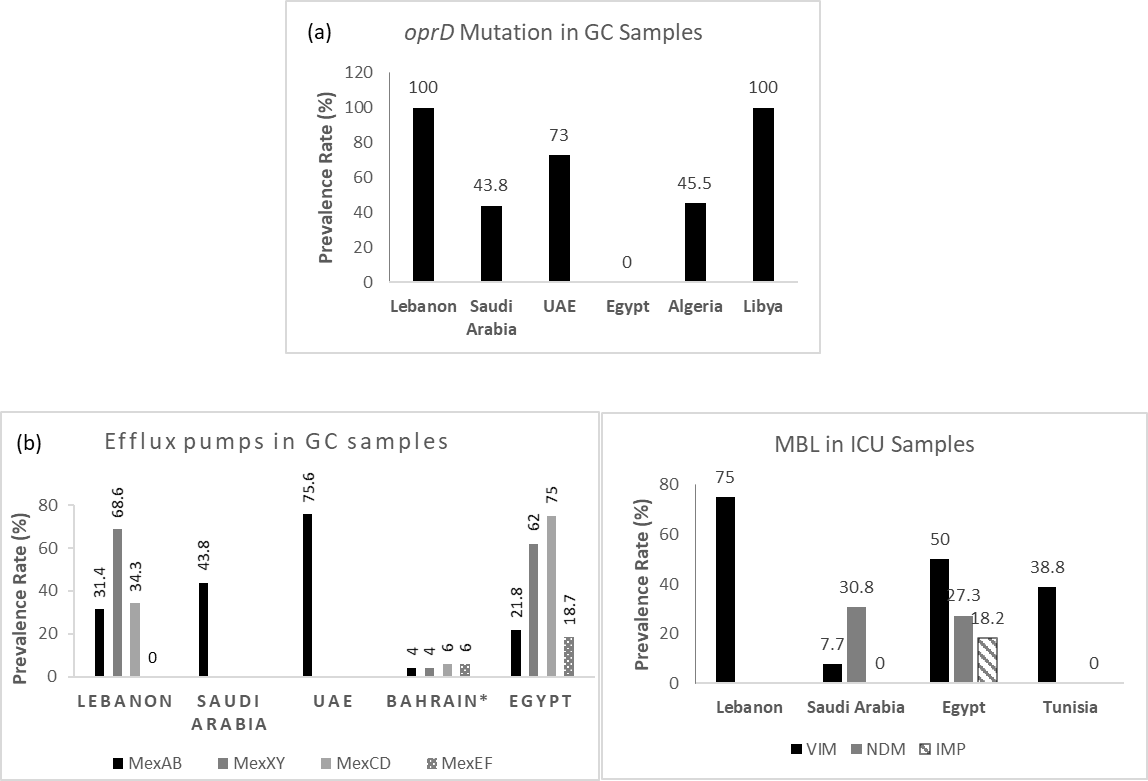

Supplement: FIG S3 [file msphere.00202-21-sf003.docx]
