# Supplementary material for: Epidemiology of Multidrug-Resistant Pseudomonas aeruginosa in the Middle East and North Africa Region
Source: mSphere. 2021 May 19;6(3):e00202-21. doi: 10.1128/mSphere.00202-21 (PMC8265635; doi:10.1128/mSphere.00202-21)
Supplement: Supplementary file 2 [file msphere.00202-21-sf002.docx]

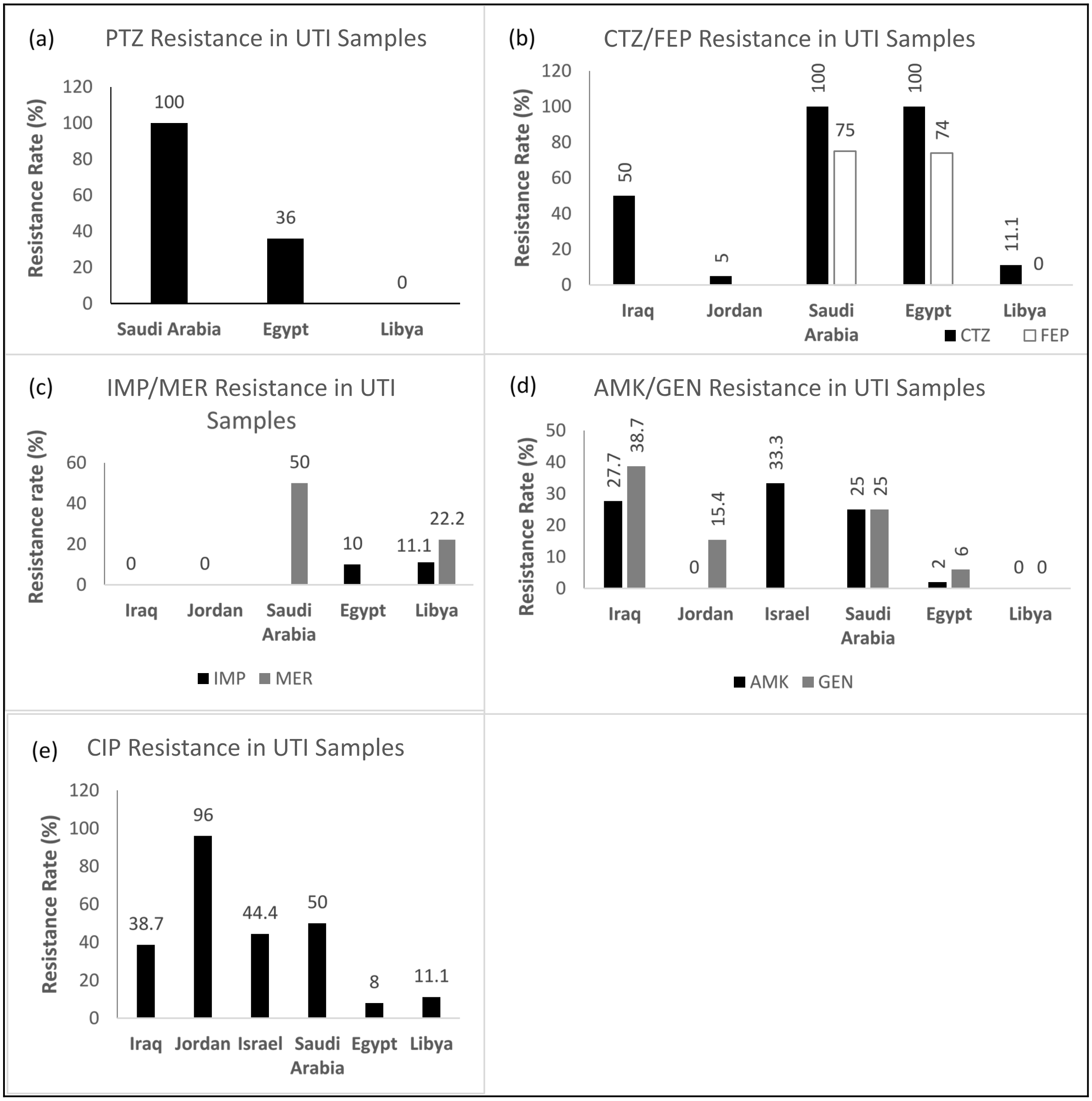
(a)
PTZ Resistance in UTI Samples
Resistance Rate (%)
120
100
80
60
40
20
0
100
36
0
Saudi Arabia
Egypt
Libya
(b)
CTZ/FEP Resistance in UTI Samples
Resistance Rate (%)
120
100
80
60
40
20
0
50
5
100
75
100
74
11.1
0
Iraq
Jordan
Saudi Arabia
Egypt
Libya
CTZ
FEP
(c)
IMP/MER Resistance in UTI Samples
Resistance rate (%)
60
40
20
0
0
0
50
10
11.1
22.2
Iraq
Jordan
Saudi Arabia
Egypt
Libya
IMP
MER
(d)
AMK/GEN Resistance in UTI Samples
Resistance Rate (%)
50
40
30
20
10
0
27.7
38.7
0
15.4
33.3
25
25
2
6
0
0
Iraq
Jordan
Israel
Saudi Arabia
Egypt
Libya
AMK
GEN
(e)
CIP Resistance in UTI Samples
Resistance Rate (%)
120
100
80
60
40
20
0
38.7
96
44.4
50
8
11.1
Iraq
Jordan
Israel
Saudi Arabia
Egypt
Libya
